# New Insights into the Role of the Locus Coeruleus-Noradrenergic System in Memory and Perception Dysfunction

**DOI:** 10.1155/2017/4624171

**Published:** 2017-11-09

**Authors:** O. Eschenko, P. B. Mello-Carpes, N. Hansen

**Affiliations:** ^1^Max Planck Institute for Biological Cybernetics, Tübingen, Germany; ^2^Stress, Memory and Behaviour Lab-Neurochemistry Lab, Federal University of Pampa, Uruguaiana, Brazil; ^3^Department of Psychiatry, University of Bonn, Sigmund Freud Str. 25, 53127 Bonn, Germany

From an evolutionary perspective, perception and memory are fundamental capacities of living beings providing adaptive behavior that is necessary for survival in ever-changing environment. Conscious and unconscious perception is generated by the interaction between many brain regions following the transduction of sensory stimuli. Sensory processing leads to cortical integration of the sensory impressions that are eventually converted to memory representations; the efficiency of sensory integration and memory retrieval underlies adaptive behavior. Everyday activities as simple as, for example, drinking water could be differentially perceived depending on an individual's emotional and arousal state. The contextual, environmental, and emotional importance of the perceived stimuli determines if the subjective experiences are subsequently remembered.

The locus coeruleus (LC) is the small nucleus in the dorsal pons comprising the main source of noradrenaline (NA) in the forebrain via its diffuse projections [1]. The LC's noradrenergic neurons are activated to aversive, rewarding, or other salient stimuli, as well as during transition from sleep to wakefulness [2–4]. Following LC activation, NA is simultaneously released from the LC terminals in the multiple brain regions [1, 5] and facilitates both local and long-range network processing as well as the experience-triggered synaptic plasticity [6–8].

In general, cognitive flexibility and adaptive behavior greatly benefit from the fact that sensory experiences, once generated by a sensory-driven network, are consequently stored within a memory supporting network with the hippocampal formation as a key element. Moreover, sensory representations are integrated with the contextual and emotional information and the retrieval of these stored associations facilitates behavioral response selection. Both the perception and the memory of events are modulated by activation of adrenoreceptors. The LC contributes to the manifold of mechanisms underlying perception and experience-induced plasticity by NA-dependent regulation of the neuronal excitability or alteration of the signal to noise ratio [6, 7, 9].

In this synopsis of the special issue, we initially focus on the role of LC for declarative memory that comprises memory about events (episodic memory) or facts (semantic memory) and mainly relies on the hippocampal formation [10]. In vivo and in vitro studies showed that a high-frequency microstimulation of the LC facilitates cellular processes related to memory formation such as synaptic long-term depression and/or potentiation in the hippocampus [11, 12]. Besides, the activation of the LC-NA system is known to facilitate memory retrieval [13] and seems to be involved in memory persistence [14].

We thus asked the following.

1. How is the LC involved in the hippocampus-dependent memory at the synaptic and microcircuit level? N. Hansen has chronologically reviewed the latest literature addressing the role of NA modulation for different stages of information processing such as encoding, consolidation, retrieval, and reconsolidation. He focuses on studies that support the role of LC in promoting hippocampal long-term plasticity and memory storage. Furthermore, he elucidates the critical nodes, such as the amygdala and prefrontal cortex, within a large-scale memory supporting network, whose activity in turn is modulated by NA. Finally, he proposes that the persistency of declarative memory is primed by the LC activity. M. Yamasaki and T. Takeuchi explain in their article how LC activity influences synaptic processes underlying memory consolidation in the hippocampus. They provide electrophysiological, immunohistochemical, and optogenetic evidence for the dopamine D1/5 receptor's involvement in the persistence of hippocampal synaptic plasticity and in the behavioral expression of memory. Furthermore, they discuss the role of the LC-dopamine system in mediating the environmental novelty signal and novelty-associated memory augmentation.
2. Is the LC-NA system also involved at the macrocircuit level? Does NA mediate the optimization of cognition and behavior ensuring cognitive and behavioral flexibility or enhanced memories? To answer this question, C. Guedj et al. investigated cortical gamma oscillations and tested the hypothesis that the coherence in gamma rhythm drives the reorganization of brain networks. According to the theory of Aston-Jones [15], NA release increases the neural gain. C. Guedj et al. suggested that the neural gain is modified by local NA release leading to the amplitude increase of gamma oscillations. This, yet hypothetic, mechanism may enhance neuronal communication and thus optimize functioning of the long-range brain networks. However, this hypothesis and proposed mechanism of the LC-mediated regulation of the brain network dynamics await the direct experimental evidence from electrophysiological studies.
3. How does the LC contribute to associative learning? This fundamental question addresses another type of memory: the implicit memory that involves associative and nonassociative learning. The cerebellum and the amygdala are critical for associative learning. M. R. Ehlers and R. M. Todd pursued the attention-related activity of LC and proposed that the LC-NA system is important for generating selective attention through associative learning in order to prioritize relevant environmental information. Specifically, they address a difference in NA availability among ADRA2b polymorphisms and make a link to psychopathology by discussing possible determinants for the development of attentional biases. They report how the LC-NA system influences aversive and appetitive conditioning in the course of associative learning and relate their findings to psychiatric disorders such as anxiety, depression, and addiction.
4. In the next section, we attempt to integrate the knowledge about the LC involvement in memory mechanisms at the synaptic, microcircuit, and network level in the context of human psychiatric disorders associated with memory and perception dysfunction. O. Borodovitsyna et al. reviewed the role of the LC-NA system in the pathophysiology of neurological disorders such as Parkinson's disease, neuropsychiatric disorders such as Alzheimer's disease, and psychiatric disorders such as attention deficit and hyperactive disorder and schizophrenia. They provide an elaborate overview on relating the functional changes in the LC-NA system with the cognitive symptoms associated with these disorders. Understanding the role of LC in cognitive dysfunction might lead to novel approaches restoring the LC function that might in turn help in developing better treatment for these patients. The article by T. Chalermpalanupap et al. pointed out the potential role of hyperphosphorylated tau protein in the LC as a sign of neurodegeneration and progression of Alzheimer's disease. Reviewing data obtained using different animal models, they question if the hyperphosphorylated tau protein accumulates in LC neurons, impairs the LC function, and also spreads throughout the brain from the NA release sites when the LC is phasically firing; answering these questions could generate new treatment strategies.

Taken together, in this special issue, we provide strong and diverse evidence for the crucial involvement of the LC-NA system in perception, memory, and behavior. Finally, the role of LC in pathophysiology of human brain disorders associated with memory and perception dysfunction is delineated, and potential starting points for treatment strategies are highlighted.

*O. Eschenko* *P. B. Mello-Carpes* *N. Hansen*
